# CD4^+^CD28^null^ T Cells are related to previous cytomegalovirus infection but not to accelerated atherosclerosis in ANCA-associated vasculitis

**DOI:** 10.1007/s00296-016-3643-8

**Published:** 2017-01-13

**Authors:** Marjan C. Slot, Abraham A. Kroon, Jan G. M. C. Damoiseaux, Ruud Theunissen, Alfons J. H. M. Houben, Peter W. de Leeuw, Jan Willem Cohen Tervaert

**Affiliations:** 1 Department of Clinical and Experimental Immunology, University Hospital Maastricht, Maastricht, The Netherlands; 2 Department of Vascular Medicine, University Hospital Maastricht, Maastricht, The Netherlands; 3 Department of Internal Medicine, VU Medical Center, De Boelelaan 1117, 1081 HV Amsterdam, The Netherlands

**Keywords:** ANCA, Vasculitis, Atherosclerosis, CD4^+^CD28 T cells, Cytomegalovirus, Intima-media thickness

## Abstract

Previous studies have suggested an increased risk for cardiovascular events in antineutrophil cytoplasmic antibodies (ANCA)-associated vasculitis (AAV). We analyzed the presence of atherosclerotic damage in patients with AAV in relation to the presence of CD4^+^CD28^null^ T cells and antibodies against cytomegalovirus (CMV) and human Heat-Shock Protein 60 (hHSP60). In this cross-sectional study, patients with inactive AAV were compared with healthy controls (HC). Carotid intima-media thickness (IMT) and aortic pulse-wave velocity (PWV) were measured. In addition, CD4^+^CD28^null^ T cells, anti-CMV, and anti-hHSP60 levels were determined. Forty patients with AAV were included. Patients’ spouses were recruited as HC (*N* = 38). CD4^+^CD28^null^ T cells are present in patients with AAV in a higher percentage (median 3.1, range 0.01–85) than in HC (0.28, 0–36, *P* < 0.0001). No significant difference in IMT (mm) between patients and controls was detected (mean 0.77 ± standard deviation 0.15 and 0.73 ± 0.11, respectively, *P* = 0.20). PWV standardized for MAP was increased in AAV patients (9.80 ± 2.50 m/s, compared to 8.72 ± 1.68 in HC, *P* = 0.04). There was a strong association between a previous CMV infection and the presence and percentage of CD4^+^CD28^null^ T cells (0.33 vs 13.8, *P* < 0.001). There was no relationship between CD4^+^CD28^null^ T cells and/or a previous CMV infection and IMT or PWV. There was no relation between anti-hHSP60 and CD4^+^CD28^null^ T cells. Increased PWV values suggest atherosclerotic damage in patients with AAV. Plaque size, as determined by IMT, did not differ. CD4^+^CD28^null^ T cells are increased in AAV and related to the previous CMV infection.

## Introduction

Small vessel vasculitides, such as granulomatosis with polyangiitis (GPA) and microscopic polyangiitis (MPA), are strongly associated with ANCA, which are either directed to myeloperoxidase (MPO) or proteinase 3 (PR3) [1, 2]. These diseases can occur in any organ system, but the respiratory tract and the kidneys are most frequently involved. Since the introduction of cyclophosphamide and prednisolone as the standard treatment, survival has improved dramatically from less than 20% at 1 year reported in 1958 [3] to at least 60% 5-year survival reported in the past 20 years [4].

With prolonged survival, patients may experience the long-term sequelae as a result of their vasculitis or its treatment [5–7]. Several studies have shown that in the long-term follow-up, cardiovascular disease is a major cause of mortality in patients with ANCA-associated vasculitis (AAV) [8–12]. Furthermore, two studies have shown that the annual incidence of myocardial infarction in patients with AAV is much higher than in the background population [13, 14].

Atherosclerosis is now widely regarded as an inflammatory disease [15], and chronic inflammation seems to be important in the progression of atherosclerosis [16, 17]. Several studies demonstrated that signs of atherosclerosis are apparent in patients with ANCA-associated vasculitis (AAV) (reviewed in [11]). Endothelial dysfunction, the earliest stage of atherosclerosis [15], is present during active disease in patients with primary systemic vasculitis [18]. Furthermore, antibodies against oxidized low-density lipoprotein (oxLDL), a marker for progression of atherosclerosis [19], are also present in active vasculitis [20]. Antibodies against human Heat-Shock Protein 60 (hHSP60) are another marker elevated in both atherosclerosis and vasculitis [21, 22].

In patients with unstable angina and myocardial infarction, the expansion of an unusual subset of CD4^+^ T cells has been described [23]. The lack of the CD28 molecule on these cells suggests repeated antigenic exposure, with cytomegalovirus (CMV) and hHSP60 as candidate antigens [24, 25]. CD4^+^CD28^null^ T cells have acquired a cytotoxic phenotype, with expression of perforin, granzyme B, and killer cell immunoglobulin-like receptors with loss of CD25, and have been shown to be cytotoxic to endothelial cells (reviewed in [26]). Interestingly, these cells have been found in GPA as well [27]. These T cells may have a role in accelerating the atherosclerotic process and/or destabilization of plaques in patients with AAV. Furthermore, an increase in IMT was described in patients with rheumatoid arthritis who had an increased percentage of CD4^+^CD28^null^ T cells [28]. In this study, we hypothesized that the presence of circulating CD4^+^CD28^null^ T cells is related to the presence of atherosclerotic damage in patients with AAV. Furthermore, we hypothesized that antibodies against CMV and hHSP60 were related to the presence of CD4^+^CD28^null^ T cells in AAV and related to the presence of atherosclerosis.

## Materials and methods

### Study design

A cross-sectional study for the presence of accelerated atherosclerosis in patients with AAV.

### Study population

All consecutive patients with AAV, who visited the outpatient clinic of our hospital and were willing to participate, were included in this study. All patients fulfilled the disease definitions for GPA, MPA, and Churg Strauss syndrome as proposed by the Chapel Hill Consensus Conference [29]. Only patients with inactive disease, as evaluated by the Birmingham Vasculitis Activity Score (BVAS) [30], were included, since an influence of active disease on the study parameters cannot be excluded. However, patients with active disease could be included at a later time point when disease was in remission. Patients’ spouses were recruited as control subjects to have an approximate match for age and traditional risk factors. The study was performed in accordance with the Declaration of Helsinki. The local medical ethics committee (MEtC, university hospital Maastricht, Maastricht) approved of the study. Written informed consent was obtained from each person.

All persons were assessed for the traditional risk factors for atherosclerosis, previous and current immunosuppressive medication use, and hsCRP.

### Laboratory analysis

Anti-CD3, anti-CD4, and anti-perforin monoclonal antibodies were purchased from Becton Dickinson (Franklin Lakes, NJ). Anti-CD28 monoclonal antibodies were purchased from Beckman Coulter (Fullerton, CA). The simultaneous presence of CD3, CD4, CD28, and perforin was evaluated on peripheral blood mononuclear cells using four-color flow cytometry (FACSCalibur, BD) and analyzed using the CellQuest software (BD). The cut-off value for percentage of CD4^+^CD28^null^ cells in our lab is established at 5%. In addition, we tested the presence of CD25 and killer cell immunoglobulin-like receptors (KIR2DS1 and KIR2DS2) (BD).

Antibodies against hHSP60 were determined as described previously [21]. Results are expressed as anti-hHSP60 levels in arbitrary units (AU)/mL. The cut-off value for this test was previously established at 80 AU/mL [21].

Anti-CMV antibodies were determined using the axSYM microparticle enzyme immunoassay (Abbott Laboratories, Abbott Park, IL). Results are expressed as anti-CMV levels in AU/mL. The cut-off value for this test is 15 AU/mL.

### Measurement of the intima-media thickness

The carotid intima-media thickness (IMT) was determined in the common carotid artery (CCA) segment 10–20 mm proximal to the tip of the flow divider. B-mode images of the IMT were acquired for the predefined 10 mm segment of the right and left CCA at defined interrogation angles using Meijers Arc. The standard images were obtained from four angles at each side. The mean IMT of the far walls was determined from a single enddiastolic B-mode image at each interrogation angle for both the right and left CCA with an automated edge-tracking method (M’Ath version 2.0.1.0, METRIS, Paris, France). In addition, the largest IMT value in the predefined segment was noted for both the left and right CCA.

### Measurement of the pulse-wave velocity

Pulse pressure waveforms were recorded from the radial and femoral artery using the Complior®SP (Colson, France). Pulse-wave velocity was then calculated between the carotid and femoral artery (representing the aortic tract), and the carotid and radial artery (representing the brachial tract). The average of ten measurements was used. As PWV is related to blood pressure, we also corrected PWV for the mean arterial pressure (MAP) [31]. MAP was calculated as diastolic pressure + 0.412 × pulse pressure [31]. MAP was standardized at 98, as this was the observed MAP in AAV patients (MAP-corrected PWV).

### Statistical analyses

All data are presented as mean ± standard deviation unless stated otherwise. Continuous data between groups were compared using Student’s *t* test and Mann–Whitney test when appropriate. The presence of antibodies in patients and controls was compared using Fisher’s exact test, obtaining Odds Ratios, and 95% confidence intervals were appropriate. Linear regression analysis was performed with percentage of CD4^+^CD28^null^ T cells and IMT as dependent variables and step-by-step exclusion of non-significant independent variables. Univariate analyses were performed with GraphPad Prism version 4.00, GraphPad Instat software package version 2.04a (both GraphPad Software Inc., San Diego, CA). Multivariate analysis was performed with SPSS 22.0 (IBM Corporation, New York, NY). A two-sided *P* value < 0.05 was considered to indicate statistical significance.

## Results

### Patient characteristics

Forty patients with AAV and 38 healthy controls (HC) were included. Mean age was 56.4 ± 10.5 in patients and 54.4 ± 9.7 in HC. Fifty-five percent of the patients were male compared to 45% of HC. The traditional risk factors for atherosclerosis differed between patients and controls as presented in Table 1. In addition, patients had significantly higher CRP levels than HC (8.0 and 2.2, respectively, *P* = 0.0002), even though only patients in clinical remission had been selected.

**Table 1:** Clinical characteristics and risk factors in patients and healthy controls

	Patients (*N* = 40)	Controls (*N* = 38)	*P* value
Age, years	56.4 ± 10.5	54.4 ± 9.7	0.38
Male	22 (55)	17 (45)	0.50
Risk factors			
Smoking			
Ever	24 (60)	18 (47)	0.36
Pack years	13.5 ± 18.1	12.0 ± 18.2	0.53
BMI	27.8 ± 5.1	25.7 ± 3.6	0.04
DM	5 (13)	1 (2.6)	0.20
Current hypertension	23 (58)	8 (21)	0.001
Systolic	130 ± 18	138 ± 18	0.04
Diastolic	76 ± 10	82 ± 10	0.01
MAP	98 ± 12	105 ± 12	0.01
Total cholesterol	5.85 ± 0.97	5.86 ± 0.99	0.96
HDL	1.49 ± 0.33	1.55 ± 0.40	0.48
LDL	3.61 ± 1.01	3.57 ± 0.91	0.85
Triglycerides	2.09 ± 1.23	1.63 ± 0.87	0.06
Family history	10 (25)	14 (37)	0.22
CRP level^a^	3.6 (0.37–47.3)	1.1 (0.15–9.5)	0.0002

Values are given as mean ± standard deviation or *N* (%)

*BMI* Body Mass Index, *DM* diabetes mellitus, *MAP* mean arterial pressure, *HDL* high-density lipoprotein, *LDL* low-density lipoprotein, *CRP* C-reactive protein

^a^Median (range)

Median disease duration was 2.6 years (range, 0.6–16.3 years, Table 2). Twenty-one patients had one or more relapses. All but one patient had used prednisolone; 34 patients had used cyclophosphamide and 36 patients had used azathioprine.

**Table 2:** Disease characteristics in ANCA-associated vasculitis

Characteristic	AAV (*N* = 40)
Disease duration, years	2.6 (0.6–16.3)
ANCA specificity	
PR3	23 (58)
MPO	10 (25)
Both	1 (3)
None	6 (15)
Disease subtype	
GPA	25 (63)
MPA	7 (18)
Churg–Strauss syndrome	4 (10)
Renal-limited vasculitis	4 (10)
Renal involvement	18 (45)
Cumulative medication	
Prednisolone, g	7.6 (0–80.2)
Cyclophosphamide, g	18.0 (0–302)
Azathioprine, g	34.2 (0–303)
Number of relapses	1 (0–5)
Number of patients with relapses	21 (53)

Values are given as median (range) or *N* (%)

*AAV* ANCA-associated vasculitis, *ANCA* antineutrophil cytoplasmic antibodies, *PR3* proteinase 3, *MPO* myeloperoxidase, *GPA* granulomatosis with polyangiitis, *MPA* microscopic polyangiitis

### Higher percentage of circulating CD4^+^CD28^null^ T cells in ANCA-associated vasculitis

**Fig. 1:**
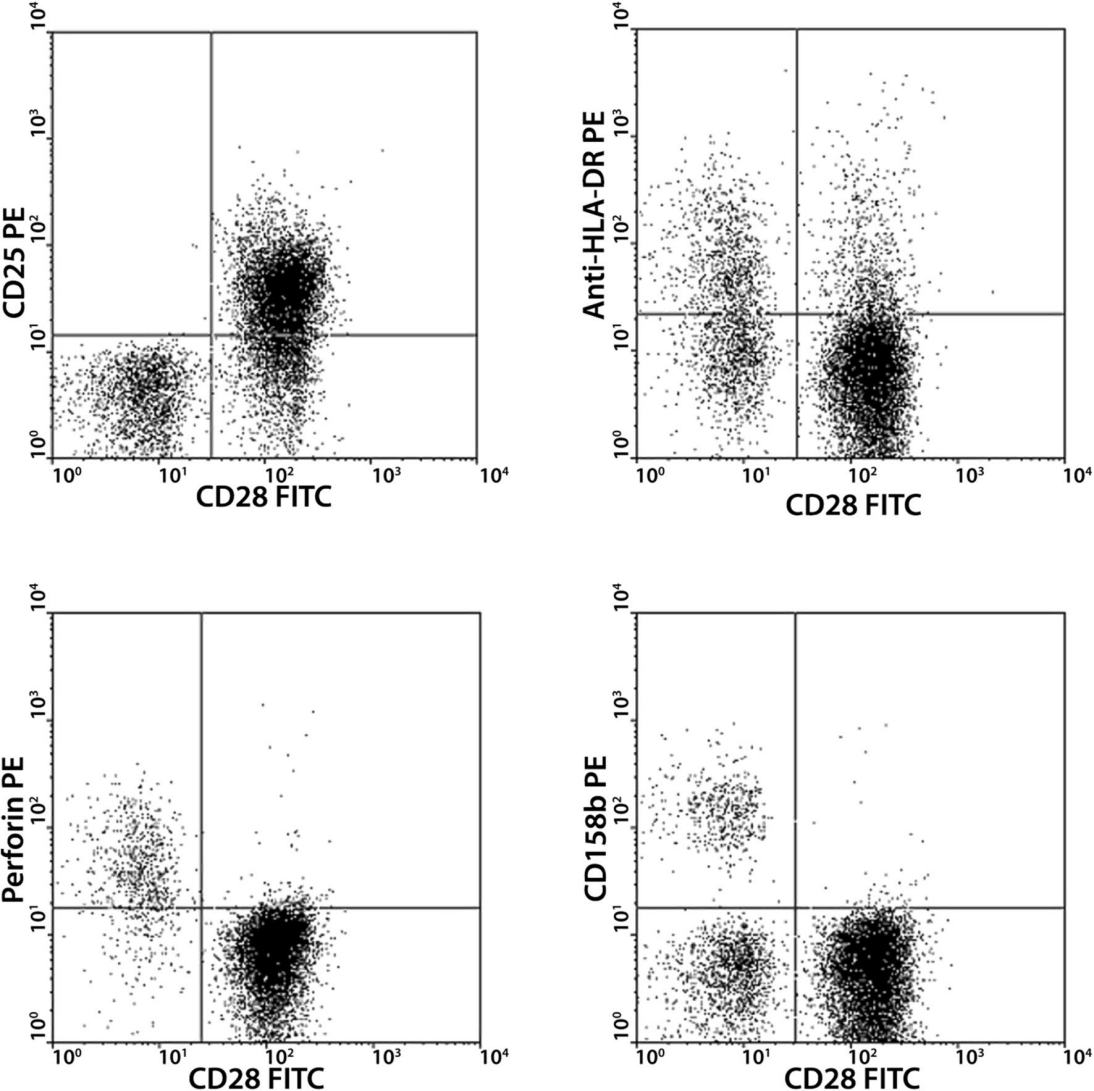
FACS analysis of CD4^+^CD28^null^ T cells. CD4^+^CD28^null^ T cells lack expression of CD25 (*upper left*). On the other hand, they are positive for perforin (*lower left*). Subsets of CD4^+^CD28^null^ T cells express HLA-Dr (*upper right*) and CD158b (a marker for killer cell immunoglobulin-like receptors, *lower right*)

In patients with AAV, the percentage of circulating CD4^+^CD28^null^ T cells was significantly higher (median 3.1%, range 0.01–85) than in HC (median 0.28%, range 0–36, Table 3). Interestingly, the absolute number of CD4^+^CD28^null^ T cells did not differ between patients and controls (Table 3). Additional FACS analysis showed that CD4^+^CD28^null^ T cells were CD25 negative, perforin positive, and to some extent positive for KIR2DS1 and KIR2DS2 (Fig. 1).

**Table 3:** Laboratory evaluation

Test	AAV patients (*N* = 40)	HC (*N* = 38)	*P*
Flow cytometry			
% CD4^+^CD28^null^ T cells	3.1 (0.01–85)	0.3 (0–36)	0.003
CD4^+^CD28^null^ cells/µL	9.8 (0.02–377)	1.5 (0–321)	0.68
ELISA			
Anti-hHSP60 (U/L)	19 (0–256)	18 (0–491)	0.98
Positive, %	6 (15)	5 (13)	1.0
Anti-CMV (U/L)	176 (0–250)	5 (0–250)	0.20
Positive, %	24 (60)	18 (47)	0.36

Values are given as median (range) or *N* (%)

*AAV* antineutrophil cytoplasmic-antibody-associated vasculitis, *HC* healthy controls, *OR* Odds ratio, *95% CI* 95% confidence intervals, *ELISA* enzyme-linked immunosorbent assay, *anti-hHSP60* antibodies against human Heat-Shock Protein 60, *anti-CMV* antibodies against cytomegalovirus

### CD4^+^CD28^null^ T cells in AAV are related to anti-CMV but not to anti-hHSP60 antibodies

**Fig. 2:**
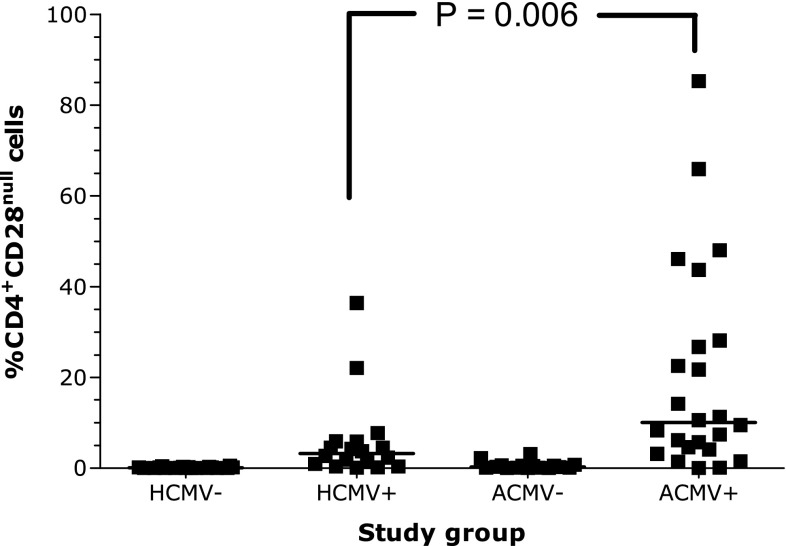
CD4^+^CD28^null^ T cells in healthy controls and vasculitis patients. CD4^+^CD28^null^ T cells are relatively more often present in patients with ANCA-associated vasculitis (AAV) who have had a cytomegalovirus (CMV) infection (ACMV+). However, they are also present in healthy controls with a previous CMV infection (HCMV+). In AAV patients and controls without a previous CMV infection, CD4^+^CD28^null^ T cells are scarcely detected (ACMV− and HCMV−, respectively)

As antigenic specificity of CD4^+^CD28^null^ T cells has been described to be for hHSP60 and CMV, we determined the relationship between antibodies against these antigens and the presence of CD4^+^CD28^null^ T cells. Prevalence of antibodies against hHSP60 and CMV was comparable between patients and controls (Table 3). We could not detect an association between antibodies against hHSP60 and the presence or percentage of CD4^+^CD28^null^ T cells. However, there was a strong association between a previous CMV infection and the presence and percentage of CD4^+^CD28^null^ T cells (0.33 vs 13.8, *P* < 0.001). Interestingly, AAV patients with a previous CMV infection had a significantly higher percentage of CD4^+^CD28^null^ T cells than HC with a previous CMV infection (Fig. 2). This association was confirmed with multiple regression analysis (HC vs ANCA, *P* = 0.016; previous CMV infection, *P* < 0.0001).

### Carotid intima-media thickness is not increased in patients with ANCA-associated vasculitis

Both the left and the right common carotid arteries were evaluated for the presence of early atherosclerotic damage. Results of the IMT measurements are presented in Table 4. Patients with AAV did not have a significantly higher IMT compared to HC (0.77 ± 0.15 and 0.73 ± 0.11 mm in the right CCA, respectively, *P* = 0.20). As the IMT was not always homogenous in patients with AAV, the maximum IMT value was additionally determined; these also did not differ between patients and controls (Table 4). In multiple regression analysis, age (*P* < 0.001), BMI (*P* = 0.009), and CRP level (*P* = 0.16) were independent predictors of IMT, but AAV was not.

**Table 4:** Vascular tests in patients and healthy controls

	Patients	Controls	*P* value
IMT			
Right, mean	0.77 ± 0.15	0.73 ± 0.11	0.20
Right, max	1.027 ± 0.20	0.98 ± 0.13	0.22
Left, mean	0.73 ± 0.12	0.72 ± 0.11	0.59
Left, max	0.96 ± 0.18	0.96 ± 0.14	0.99
PWV			
Femoral	9.73 ± 2.76	9.27 ± 2.15	0.44
Femoral MAP-corrected PWV	9.77 ± 2.41	8.71 ± 1.71	0.04
Radial	8.85 ± 1.38	8.47 ± 1.88	0.32
Radial MAP-corrected PWV	8.86 ± 1.41	7.95 ± 1.77	0.02

All values are mean ± standard deviation

*IMT* intima-media thickness (mm), *PWV* pulse-wave velocity (m/s)

### Pulse-wave velocity is increased in patients with ANCA-associated vasculitis

To determine vascular elasticity, pulse-wave velocity was measured over the carotid-femoral and carotid-radial arterial segments. Since PWV is related to the blood pressure, and blood pressure was significantly higher in our HC, we corrected PWV in both groups for MAP and compared both PWV and MAP-corrected PWV. Femoral PWV was comparable between AAV patients and controls, as was radial PWV (Table 4). However, when PWV values were corrected for MAP, femoral PWV was higher in AAV patients (9.77 ± 2.41 m/s, compared to 8.71 ± 1.71 m/s in HC, *P* = 0.04). In addition, radial MAP-corrected PWV was higher in patients with AAV (Table 4).

### CD4^+^CD28^null^ cells and previous CMV infection not associated with increase in IMT or PWV

We could not detect a difference in IMT between patients with >5% CD4^+^CD28^null^ T cells and patients with <5%. In addition, there was no correlation between IMT and % CD4^+^CD28^null^ T cells. Furthermore, PWV measurements also did not differ between patients with a high or low percentage of CD4^+^CD28^null^ T cells. In addition, we could not detect a difference between patients and controls with or without a previous CMV infection.

## Discussion

In the present study, we determined the presence of early atherosclerotic damage, comparing carotid IMT and femoral and radial PWV between AAV patients and healthy age- and sex-matched volunteers. No difference in IMT between patients and controls was detected. PWV standardized for MAP, however, was increased in AAV patients compared to healthy controls. Furthermore, CD4^+^CD28^null^ T cells were found to be present in patients with AAV in a higher percentage compared to controls and were found to be related to a previous CMV infection. No direct relationship, however, between CD4^+^CD28^null^ T cells and IMT and/or PWV was found.

Patients with AAV who survive the acute phase of their vasculitis often die of cardiovascular disease in long-term follow-up [8–12]. In addition, patients with AAV have a higher incidence of myocardial infarction than their background population [13, 14]. The increased incidence of myocardial infarction in AAV without an increase in IMT and only marginal differences in PWV raises the possibility that it is not plaque size but plaque stability that may be different between AAV patients and healthy subjects. Liuzzo and colleagues reported the presence of CD4^+^CD28^null^ T cells in patients with unstable angina or myocardial infarction [23]. CD4^+^CD28^null^ T cells are resistant to apoptosis and the percentage of these cells remains remarkably stable during follow-up [32]. Furthermore, these cells are cytotoxic for endothelial cells [33] and produce large amounts of interferon γ [33], both factors that are relevant for plaque instability. Interestingly, these cells had only invaded the “culprit plaques”, i.e., plaques that have ruptured and seemed to have caused myocardial infarction. Patients with AAV have an increased percentage of circulating CD4^+^CD28^null^ T cells. Therefore, we postulate that although plaque size is comparable between patients and controls, the increase in CD4^+^CD28^null^ T cells in AAV patients may result in an increased propensity for myocardial infarction. It may, therefore, be interesting to look at the presence of rupture-prone plaques in patients and controls [34]. In addition, there may be other factors involved in the occurrence of cardiovascular events in AAV patients, such as local coronary disease [35] and increased thrombogenic risk in patients with AAV [36].

The reason why CD4^+^CD28^null^ T cells are relatively increased in AAV is not yet clear. Previous studies suggested that hHSP60 and/or CMV may be candidate antigens that are recognized by these T cells [24, 25]. In our study, we could not detect an association between CD4^+^CD28^null^ T cells and the presence of anti-hHSP60 antibodies. A previous CMV infection, however, was highly associated with the presence of CD4^+^CD28^null^ T cells. The percentage of CD4^+^CD28^null^ T cells was even higher in AAV patients with a previous CMV infection compared to the percentage of these cells in healthy controls with a previous CMV infection. This may be due to (subclinical) CMV reactivation in AAV patients with a previous CMV infection, caused by the immunosuppressive therapy that is needed to control active vasculitis. The lack of the CD28 molecule on CD4^+^CD28^null^ T cells suggests repeated antigenic exposure [24], supporting this hypothesis. Interestingly, CMV infection has been associated with the presence of atherosclerosis as well [37]. In mice, CMV infection increases atherosclerosis [38].

Treatment with cytostatic therapy with cyclophosphamide and/or azathioprine may influence the development of atherosclerotic lesions [18, 39]. In patients with chronic kidney disease, treatment with immunosuppressive drugs had a positive effect on blood pressure and PWV [40]. On the other hand, an increase in body weight, hypertension, and diabetes mellitus is well-known side effects of the use of corticosteroids and also risk factors for developing atherosclerosis. In fact, a study by Wei and colleagues showed that chronic use of glucocorticoids is associated with an increase of cardiovascular events [41].

This study has some limitations. First, our group of vasculitis patients is relatively small, resulting in a low power of our study. Second, we used spouses as healthy controls, to correct for influences of lifestyle on atherosclerosis. This may, however, have resulted in only a small difference between our patients and controls. Indeed, in a study by de Groot et al. [42], middle-aged healthy controls had an average carotid IMT of 0.59 mm instead of the 0.73 mm we found in our study. Third, although we found a difference in PWV between patients and controls, PWV <12 m/s is still within the normal range. Fourth, we did not correct for the present use of medication or presence of decreased renal function, although we did exclude patients who are dialysis dependent given that these patients already are at increased risk for atherosclerosis [43].

In conclusion, we found only minor evidence of accelerated atherosclerosis in patients with ANCA-associated vasculitis when studying IMT and PWV. CD4^+^CD28^null^ T cells, however, are relatively increased in AAV and found to be related to the previous CMV infection. The occurrence of these cells may be relevant for plaque instability and responsible for the observed increased incidence of myocardial infarction in these patients.
